# Supplementary material for: Prevalence of depression, anxiety and stress and their risk and protective factors among secondary students in Rwanda during the first wave of COVID-19 pandemic
Source: PLoS One. 2024 Jul 1;19(7):e0306389. doi: 10.1371/journal.pone.0306389 (PMC11216594; doi:10.1371/journal.pone.0306389)
Supplement: Attachment — Submitted filename: Rebuttal letter to reviewers.doc [file pone.0306389.s001.doc]

**Rebuttal letter**

We wish to express our sincere appreciation to the editor and reviewer for their insightful comments. We have done our best to address all the issues raised and we hope that the final version of this manuscript is much improved due to their comments.

**Note:** This table corresponds to the manuscript with track changes. It means that page numbers correspond to the manuscript with track changes.

| **N** | **Reviewer 1** | **Reviewer’s comments** | **Page and line on which addressed** | **How comments were addressed** |
| --- | --- | --- | --- | --- |
|  | **General comments** | **Comments to the Author**  1. Is the manuscript technically sound, and do the data support the conclusions?  Reviewer #1: Yes  Reviewer #2: Yes  2. Has the statistical analysis been performed appropriately and rigorously?  Reviewer #1: Yes  Reviewer #2: Yes  3. Have the authors made all data underlying the findings in their manuscript fully available?  Reviewer #1: Yes  Reviewer #2: NO  4. Is the manuscript presented in an intelligible fashion and written in standard English?  Reviewer #1: Yes  Reviewer #2: Yes | Whole document | We really appreciate the reviewer 1 and reviewer 2 for the overall appreciation of the quality of this manuscript. |
| 1 | Reviewer #1: Minor comments | 1. What is the rational for selecting equal number of participants (128 each) from three districts (disproportional recruitment)? | Page 5, line 136 | **We apologize for this error stemming from the initial proposal before presentation to the ethical committee. In an effort to correct, we have added the following in the method section:**  ***Participants***  *Eligible participants were all students actively enrolled before the covid-19 outbreak in Rwanda on March 14, 2020, and aged between 14-24 years. Students who were seriously ill during data collection phase were excluded to ensure the accuracy and integrity of the response. A proportional stratification approach was adopted to select a sample that accurately reflected the diversity of the student population in the chosen districts. Sample size was calculated with Cochran’s formula (2004),(**), using parameters: 50% proportion (p=0.5), 95% confidence intervals (****z****=1.96), 5% margin of error (****e****=0.05) [27]****.*** *Given the historically low non-response rate (<1%) in similar Rwandan populations, an adjustment for non-response was deemed unnecessary. This decision was based on empirical evidence suggesting minimal impact of non-response bias in our context. Thus, the final sample size was set at 384 participants, considered adequate for robust statistical analysis.* |
|  |  | 2. Line 152 “Qualified health experts from the schools selected for this study…”. Are there Health experts in primary school in Rwanda?, why? | Page 5, line 149 | ***Thank for the insightful comment. We apologize for the confusion caused by the term “*Qualified health experts”*, we have corrected as follows:***  ***Data collection and procedure***  *To ensure comprehension and cultural relevance, the research instruments were translated into Kinyarwanda and back-translated to English. Following a pre-test on 5% of samples (20 participants) from non-selected schools in Gasabo district, minor adjustments were made to the survey tools based on feedback.* ***Data collection was conducted through in-person interviews by principal investigator and three research assistants (clinical psychologists), with school matrons facilitating the approach to the research participants.*** |
|  |  | 3. Language editing by native speakers | Whole document | **Thanks for the recommendation.** The manuscript grammar was improved using the Grammarly, and then proofread by Native English speaker “Pascaline Mugiraneza Munezero”. |
|  | Reviewer #1: Major comments | 1. The first wave of COVID-19 occurred in late 2019 and early 2020, however this study was conducted three years later, in 2022. Participants may not remember the exact situation during the first wave after three years. This may have an impact on the study's generalizability. | Page 17, Line 344 | **You’re right. We forget to specify that this was retrospective in the study design earlier.** The covid-19 outbreak in Rwanda was March 14, 2020, and the lockdown, when all primary and secondary schools were closed and re-opened in September 2020 after the lockdown. Our study was conducted at the beginning of 2022, meaning that was conducted around year and half later. However, as reviewer said, this would be one of the limitations which was acknowledged in the discussion section.  *Strengths and limitations*  The study’s strengths lie in its pioneering examination of the mental health outcomes among Rwandan secondary students during the COVID-19 pandemic, offering invaluable insights for the policymakers and shedding light on the pandemic's impact on the youth. Furthermore, its retrospective nature provides historical context, enriching our understanding of the evolving mental health landscape amidst the crisis. However, alongside its contributions, the study faces limitations, notably its retrospective nature, which, while offering valuable historical insights, may be constrained by the accuracy of recall among participants. Additionally, the cross-sectional design poses challenges in establishing causal relationships between pandemic-related factors and observed mental health outcomes. |
|  |  | 2. Re design all tables and use appropriate font size for texts | Page 11, line 281 | **We appreciate the reviewer for the constructive comments.**  We have strongly complied with your suggestion by adjusting the font size for texts according to the instruction for authors available from Plos one journal website. |
|  |  | 3. Make the discussion more concise, keeping the main points | Page 16, line 282 | **We appreciate the reviewer for the constructive comment. we have rigorously revised the discussion sections as follows:**  ***Discussion***  *This study explored the prevalence of mental health outcomes (depression, anxiety and stress) and their associated risk and protective factors among the students from high schools in Rwanda during the Covid-19 pandemic. Our findings indicated that 51%, 30.3%, and 67.3% of our sample reported clinically high levels of depression, stress, and anxiety, respectively. Despite the scarcity studies conducted exclusively on secondary students, the prevalence of depression, stress and anxiety in our study is lower yet comparable to those reported in the general population of Bangladesh[37], college students in Ethiopia [16] and primary and secondary school students in the Gaza Strip in Palestine [12]during the lockdown.*  *According to Abir et al.[37], approximately 64%, 87%, and 61% of the respondents in Bangladesh had clinically significant symptoms of depression, anxiety, and stress, respectively. Similarly, 77.2%, 71.8%* *and 48.5% of Ethiopian college students reported high levels of depression, anxiety and stress during the lockdown, respectively [16]. Among residents of Tepi town in Ethiopia, the prevalence of depression, anxiety, and stress symptoms was 37.7%, 39.0%, and 44.2%, respectively* [38]. *In the Gaza Strip, the majority of the students experienced anxiety (88.4%) and depression (72.1%), while more than one-third (35.7%) experienced stress[12]. However, the prevalence in the current study is significantly higher than that reported among undergraduate students in Saudi Arabia, where anxiety, depression and stress were reported at 22.2%, 25.4%, 17.9%, respectively[38]. This difference may be attributed to the varying education levels of the samples, retrospective nature of this study, and the multitude of risk factors investigated in this study.*  *As revealed in previous studies, our findings highlighted that negative mental well-being, being infected with COVID-19, direct contact with Covid-19 infected individuals[39], and experiencing symptoms of COVID-19 (sore throat, coryza, myalgia, fever and dizziness),[40], being in the 11^th^ grades or 12^th^ grades[41], exposure to domestic violence and being female [42–45]increased the odds to have depression[46], anxiety[46] and stress. Students in the 9^th^ and 12^th^ grades faced higher odds of experiencing these mental disorders, possibly due to the pressures of preparing for the national exam, and frequent exposure to social media platforms spreading rumours, and biased information about COVID-19. Additionally, the closure of schools during the COVID-19 pandemic likely exacerbated concerns over educational achievements[47]. Our study confirmed that female students were more* *prone to depression, anxiety and stress, which may be explained by differences in risk perception[42]. This finding aligns with previous research indicating that female participants are more susceptible to psychological problems during COVID-19 [43,44]. Experiencing COVID-19 symptoms [40] was significantly associated with increased odds of depression, anxiety and/or stress. The pandemic's impact on daily life, including restrictions on social interaction and financial constraints, likely contributed to psychological distress among students.*  *Interestingly, students who had one of the three mental disorders had greater odds to have another mental disorder, suggesting a comorbidity among these conditions [48]. Another significant finding was that the students from families in the second social category were more likely to suffer from these mental health issues, a result consistent with studies highlighting the impact of low-income on mental health during the pandemic [49,50]. This result could be attributed to the fact that low-income families have limited access to daily necessities and materials required for preventive procedures during the pandemic [49,50]. Contrary to previous studies [50,51], we found that students from larger families were less likely to experience mental disorders.*  *Consistent with previous research, our findings identified several protective factors against depression, anxiety, and stress among high school students in Rwanda during the COVID-19 pandemic. These include awareness of Covid-19[52], social support [53,54], being in the third Ubudehe category, positive mental health[55] and high resilience [11,56,57]. Echoing Zhang et al.[11], resilience emerges as key mediator that, alongside positive emotions can significantly enhance their coping mechanisms and overall well-being under stress (i.e. the COVID-19 pandemic), [58]. Additionally, the strong negative correlation between social support and symptoms of mental distress reinforces the importance of a supportive network during crises, highlighting its role in mitigating the psychological impacts[53,54].‏ These insights emphasize the need for integrated strategies that prioritize mental health support, resilience-building, and the fortification of social support systems to combat the adverse mental health effects of public health emergencies effectively[38].*  *Strengths and limitations*  *The study’s strengths lie in its pioneering examination of the mental health outcomes among Rwandan secondary students during the COVID-19 pandemic, offering invaluable insights for the policymakers and shedding light on the pandemic's impact on the youth. Furthermore, its retrospective nature provides historical context, enriching our understanding of the evolving mental health landscape amidst the crisis. However, alongside its contributions, the study faces limitations, notably its retrospective nature, which, while offering valuable historical insights, may be constrained by the accuracy of recall among participants. Additionally, the cross-sectional design poses challenges in establishing causal relationships between pandemic-related factors and observed mental health outcomes. Therefore, longitudinal studies and real-time data collection efforts are essential to accurately capture the pandemic's long-term effects and inform more effective interventions and support systems for youth mental health.*  ***Conclusion***  *This study underscores the high prevalence of depression, anxiety and stress among secondary school students during the COVID-19 pandemic. Identified risk factors include domestic violence, COVID-19 symptoms, co-morbidities among the mental health outcomes, gender and direct contact with infected individuals. Conversely, awareness of Covid-19, positive mental health, social support, belonging to the third Ubudehe category, and high resilience were protective. The findings call for targeted interventions by policymakers and decision-makers, emphasizing the need for widespread vaccination and the cultivation of resilience and positive mental health during crises.* |

| **N** | **Reviewer 2** | **Reviewer’s comments** | **Page and line on which addressed** | **How comments were addressed** |
| --- | --- | --- | --- | --- |
| 1 |  | 1. Your study result showed that, common associated risk factors were domestic violence, the existence of some symptoms of COVID-19 such as cough and myalgia, co-morbidities between depression, anxiety and stress, religion, being female and direct contact with the people who positively tested covid-19. How religion is associated with anxiety, stress, and depression? Is it logical? | Page 2, line 40 | Thank you very much for the insightful comment. we apologize for the grammatical error in the abstract result section, we have corrected it as follows:  ***Results****: The results indicated that slightly above half of the participants (51%, n=195) had clinically significant symptoms of depression, 30.3% (n=116) had stress and 67.3% (n=259) had anxiety. Our analyses identified several key risk factors associated with increased odds of these mental disorders. These include exposure to domestic violence, COVID-19 symptoms like cough and myalgia, eating twice per day, having one of the three mental disorders, gender, with females showing higher susceptibility, and direct contact with the people who positively tested covid-19. Conversely, protective factors such as heightened awareness about Covid-19, positive mental health, social support, eating three times, belonging to the third Ubudehe category, and a high resilience emerged as significant elements mitigating the risks of these mental health challenges within our sample. Intriguingly, religious affiliation emerged as a notable factor, with students affiliated with the Witness of Jehovah and Adventist denominations exhibited lower risks for depression and anxiety.* |
| 2 |  | 2. Line 9, at the affiliation of authors, you used code 3, as “ 3Department of Business Administration, College of Business and Economics, University of 10 Rwanda, Kigali, Rwanda”, however no author with such code on list of authors. The contribution of the author from the Department of Business Administration, College of Business and Economics, appears to be outside their discipline. It may be necessary to clarify the relevance of their contribution to the research. | Page 13, line 1753 | We sincerely appreciate the reviewer's insightful comments, which have guided us in refining the authors contributions. We apologize for this error, the author with 3 as affiliation is abbreviated as LU. She is very interested in research, and she has many certificates from social research-related training.  ***Authors’ contributions***  *MBM, JM, CUK conceived and designed the study, collected data, and participated in statistical analyses. JN contributed to and led statistical analyses, interpretation of the results, and drafted the manuscript. LU contributed to risk assessment, data cleaning, data monitoring, and project administration. CUK and JM were the study supervisors. All authors reviewed, edited, and approved the final manuscript for submission to the journal for publication.* |
| 3 | **Material and Methods** | 3. • Line 137, When substituting the values for p and 1-p in the sample size determination formula on line 137, ensure that the correct p-value is used. Double-check and correct any missing or incorrect values to ensure accuracy in the calculation.  4. While determining sample size, why non-response rate is not considered? Non-response rate is often considered when determining sample size, as it can impact the representativeness of the sample. Ignoring non-response rate could lead to biased results and affect the generalizability of the findings. | Page 5, line 136 | **We greatly appreciate the thoughtful and constructive comments provided by the reviewer**. The paragraph was improved as follows:  *Eligible participants were all students actively enrolled before the covid-19 outbreak in Rwanda on March 14, 2020, and aged between 14-24 years. Students who were seriously ill during data collection phase were excluded to ensure the accuracy and integrity of the response. A proportional stratification approach was adopted to select a sample that accurately reflected the diversity of the student population in the chosen districts. Sample size was calculated with Cochran’s formula (2004),(**), using parameters: 50% proportion (p=0.5), 95% confidence intervals (****z****=1.96), 5% margin of error (****e****=0.05) [27]****.*** *Given the historically low non-response rate (<1%) in similar Rwandan populations, an adjustment for non-response was deemed unnecessary. This decision was based on empirical evidence suggesting minimal impact of non-response bias in our context. Thus, the final sample size was set at 384 participants, considered adequate for robust statistical analysis.* |
|  |  | 6. This institutional-based, cross-sectional study was conducted from 2nd January to 4th April 2022….. The duration of the study (January to April 2022) may impact the response, as the prevalence and severity of symptoms could have changed since 2019-2020. How do you solve this problem, since the duration may affect the response? | Page 17, Line 344 | **You’re right. We forget to specify that this was retrospective in the study design earlier.** The covid-19 outbreak in Rwanda was March 14, 2020, and the lockdown, when all primary and secondary schools were closed and re-opened in September 2020 after the lockdown. Our study was conducted at the beginning of 2022, meaning that was conducted around year and half later. However, as reviewer said, this would be one of the limitations which was acknowledged in the discussion section.  *Strengths and limitations*  *The study’s strengths lie in its pioneering examination of the mental* *health outcomes among Rwandan secondary students during the COVID-19 pandemic, offering invaluable insights for the policymakers and shedding light on the pandemic's impact on the youth. Furthermore, its retrospective nature provides historical context, enriching our understanding of the evolving mental health landscape amidst the crisis. However, alongside its contributions, the study faces limitations, notably its retrospective nature, which, while offering valuable historical insights, may be constrained by the accuracy of recall among participants. Additionally, the cross-sectional design poses challenges in establishing causal relationships between pandemic-related factors and observed mental health outcomes*. |
|  |  | • Line 192, …..we found that the consistency was also good (Alpha of Cronbach, α=0.86) (Error! Reference source not found.). check table citation problem and correct it. | Whole method section | **We apologize for this error, the table for internal consistency does not exist.** Therefore, we have removed the table 5 citation. |
|  |  | It seems that prior study during pandemic <https://doi.org/10.1007/s40615-021-01195-1> not discussed. Check and discuss with your study. |  | **Thank you for the insightful comment. This important information was added to the discussion section** as follows:  *According to Abir et al.[37], approximately 64%, 87%, and 61% of the respondents in Bangladesh had clinically significant symptoms of depression, anxiety, and stress, respectively. Similarly, 77.2%, 71.8% and 48.5% of Ethiopian college students reported high levels of depression, anxiety and stress during the lockdown, respectively [16].* ***Among residents of Tepi town in Ethiopia, the prevalence of depression, anxiety, and stress symptoms was 37.7%, 39.0%, and 44.2%, respectively*** *[38].* |
|  |  | • Please add the direction for the future work based on your work and prior studies. |  | **Thank you for the constructive comment, we have added the following information after highlighting the strengths and limitations:**  *Therefore, longitudinal studies and real-time data collection efforts are essential to accurately capture the pandemic's long-term effects and inform more effective interventions and support systems for youth mental health.* |
|  |  | • Line 389, <> I don’t think that putting reference for this declaration is important. I suggest to remove reference. | Page7, line 215 | **Thank you for the constructive comment, the reference was removed:**  ***Ethical considerations***  *This study adhered to the Helsinki Declaration principles, securing the approval from the Institutional Review Board (n^o^: 394/CMHS IRB/2021) of the College of Medicine and Health Sciences (CMHS), University of Rwanda. Permission was also obtained from the Ministry of Education and participating schools. Informed consent (or assent for minors) was obtained from all participants after a thorough explanation of the study's purpose, benefits, and procedures.* |
|  |  | 4. It's important to thoroughly check the document for language, typos, grammar, and accurate interpretation of findings. This ensures the overall quality and credibility of the document. | Whole document | **Thanks for the recommendation.**  *The manuscript grammar was improved using the Grammarly, and then proofread by Native English speaker “Pascaline Mugiraneza Munezero”.* |
